# Intelligent Mobile Wireless Network for Toxic Gas Cloud Monitoring and Tracking

**DOI:** 10.3390/s21113625

**Published:** 2021-05-23

**Authors:** Mateusz Krzysztoń, Ewa Niewiadomska-Szynkiewicz

**Affiliations:** 1 Research and Academic Computer Network (NASK), Kolska 12, 01-045 Warsaw, Poland; mateusz.krzyszton@nask.pl; 2 Institute of Control and Computation Engineering, Warsaw University of Technology, Nowowiejska 15/19, 00-665 Warsaw, Poland

**Keywords:** phenomena clouds monitoring, boundary estimation, MANET, wireless sensor network, self-organization, artificial potential field, simulation

## Abstract

Intelligent wireless networks that comprise self-organizing autonomous vehicles equipped with punctual sensors and radio modules support many hostile and harsh environment monitoring systems. This work’s contribution shows the benefits of applying such networks to estimate clouds’ boundaries created by hazardous toxic substances heavier than air when accidentally released into the atmosphere. The paper addresses issues concerning sensing networks’ design, focussing on a computing scheme for online motion trajectory calculation and data exchange. A three-stage approach that incorporates three algorithms for sensing devices’ displacement calculation in a collaborative network according to the current task, namely exploration and gas cloud detection, boundary detection and estimation, and tracking the evolving cloud, is presented. A network connectivity-maintaining virtual force mobility model is used to calculate subsequent sensor positions, and multi-hop communication is used for data exchange. The main focus is on the efficient tracking of the cloud boundary. The proposed sensing scheme is sensitive to crucial mobility model parameters. The paper presents five procedures for calculating the optimal values of these parameters. In contrast to widely used techniques, the presented approach to gas cloud monitoring does not calculate sensors’ displacements based on exact values of gas concentration and concentration gradients. The sensor readings are reduced to two values: the gas concentration below or greater than the safe value. The utility and efficiency of the presented method were justified through extensive simulations, giving encouraging results. The test cases were carried out on several scenarios with regular and irregular shapes of clouds generated using a widely used box model that describes the heavy gas dispersion in the atmospheric air. The simulation results demonstrate that using only a rough measurement indicating that the threshold concentration value was exceeded can detect and efficiently track a gas cloud boundary. This makes the sensing system less sensitive to the quality of the gas concentration measurement. Thus, it can be easily used to detect real phenomena. Significant results are recommendations on selecting procedures for computing mobility model parameters while tracking clouds with different shapes and determining optimal values of these parameters in convex and nonconvex cloud boundaries.

## 1. Introduction

Modern industry produces substances whose volatilization causes a severe threat to the environment. Many of these hazardous substances form gas clouds heavier than air when accidentally released into the atmosphere. As a result, the gas cloud changes its shape, which is usually irregular, moves and covers a larger area with time. Toxic gases usually endanger humans [1]. The strategic step for conducting a situational assessment is to create a sensing system to determine a gas-covered area with a concentration greater than the safe value [2].

Research and commercial implementations in recent years have shown that self-configuring networks built from static and mobile wireless devices equipped with sensing modules can significantly enhance the capability to investigate and sense unknown environments [3]. Such networks can be successfully used to monitor phenomena, such as clouds created by heavy hazardous toxic substances [4]. Mobile devices equipped with a Central Processing Unit (CPU), radio transceiver, Global Positioning System (GPS), and gas detector can exchange data and dynamically change both the position and role in the network. However, to ensure accurate measurement and effective tracking of moving clouds, an efficient computing scheme for online trajectory planning and a reliable interconnection network of sensing devices is required. Moreover, low processing power, low-quality wireless communication, limited radio range and energy resources are significant limitations that make the implementation of such a network even more complex.

This paper presents a comprehensive approach to heavy gas cloud monitoring that can be used for disaster management and toxic spill counteraction using unmanned ground vehicles or drones. Specifically, it describes a computing scheme for creating sensing mobile ad hoc networks (MANETs) for estimating and tracking cloud boundaries and reporting the results to the central dispatcher of the system (base station) to build situational awareness. The proposed computing scheme incorporates gas dispersion modeling [5], wireless transmission modeling [6], motion modeling [7] and clustering techniques [8]. The network operation consists of three stages: (i) exploration of the sensing scene and gas cloud detection, (ii) exploration of the cloud and its boundary detection, (iii) tracking the evolving and moving cloud. The focus is on mobility modeling and adaptation of the boundary tracking algorithm to various shapes of gas clouds. Our approach’s main originality is three algorithms for online network node displacement calculations in the mentioned phases of a gas cloud monitoring process. New positions of sensing devices are computed, solving the optimization problems with differentiable real-valued performance measures, which are viewed as energy. To improve the sensing network’s long-term stable operation, multi-hop communication with the base station (BS) is implemented. Thus, a cooperative and coherent network comprising fully autonomous smart sensors that can operate without any external fixed communication infrastructure is proposed. It is assumed that sensing devices can constantly exchange data with their neighbors and finally transmit measurements to BS. There is no central control in the system, and all nodes make autonomous decisions based on sensor readings and data provided by their neighbors. Multi-hop communication with BS reduces the energy costs and communication delays compared to direct communication between BS and a given sensor node. It enables the system to monitor vast areas covered with toxic substances and tracking clouds located at a considerable distance from the base station. In general, it improves system scalability, resilience and reliability.

In most sensing systems described in the literature, an exact value of gas concentration and its gradient are used to calculate the expected positions of sensors. The concentration gradient indicates the boundary of a cloud. It is possible to assess the gas concentration in the whole sensing scene using adequate gas dispersion models and a series of measurements. Due to negative buoyancy, heavy gas clouds’ behavior differs from typical pollution clouds [9]. The dynamics of changes are minor. Despite this, the monitoring and tracking of such phenomena is a challenging problem. In the case of gases heavier than air, even slight changes to the terrain seriously influence the concentration gradient. The extent and shape of the cloud depend on current atmospheric conditions, including wind strength. Unfortunately, most gas dispersion models do not consider changes to the terrain and obstacles [10]. It is assumed that the cloud moves over a flat area and ignores local perturbations of the gas concentration. It results in a simplified situation where the gas concentration decreases towards the cloud boundary. In reality, this situation does not usually occur. Moreover, heavy gas clouds are characterized only by a negative vertical concentration gradient, which does not help detect the cloud boundary. The vertical gradient causes turbulent gas mixing, further disturbing the horizontal gas concentration regularity of the distribution. Therefore, methods that determine the direction of motion based on the exact value of the measured concentration can give outstanding results in simulators and fail in the real world. In our system, sensor readings are reduced to two values, namely the gas concentration below or greater than the safe value, to decrease its sensitivity to the accuracy of gas concentration measurements.

To sum up, this work’s main contribution is to present the general overview of the system for detection of a heavy gas cloud in a working space *W*, detection of its boundary, and its estimation and tracking. The algorithms for motion planning and cooperation of the sensing devices used at each stage of the gas cloud exploration are presented and discussed. The system can be used for disaster management using unmanned vehicles or drones equipped with sensors. The data collected by the sensors and transmitted to the emergency management center will allow creating emergency awareness, supporting the evacuation of people from the endangered area, supporting the rescue teams conducting operational activities, and neutralizing a toxic spill.

The presented results extend the work described in [11]. The paper [11] addresses the problem of cloud boundary detection and estimation. In this paper, a brief overview of algorithms for cloud boundary detection and estimation is provided. The attention is paid to the most challenging task, namely, a boundary of slowly moving cloud tracking. The main contribution is a novel algorithm for convex and nonconvex gas cloud boundary tracking and five procedures for tuning the algorithm’s parameters. An exhaustive simulation study that shows the effectiveness of these procedures depending on the considered scenario is presented. Finally, we provide recommendations for the choice of a procedure, respectively, for a given cloud shape.

The rest of the paper is structured as follows. Section 2 presents the survey of the application of MANETs to monitor and truck phenomena clouds. Section 3 provides a formulation of the problem to be solved and the model of a network composed of smart wireless sensing devices. Section 4 describes the concept of a three-stage strategy for heavy gas cloud monitoring and tracking and computing schemes for online motion trajectories calculation. A comparative study of a few variants of procedures for tuning model parameters is presented in Section 5. Finally, Section 6 concludes the paper and highlights future research directions. The Appendix A provides a list of notations used that is standard across all of the sections.

## 2. Related Work

The problem of developing effective and valuable strategies for detecting, estimating and tracking boundaries using collaborating static and mobile sensing devices has been studied in recent years by many researchers. Various techniques have been investigated, implemented and tested through simulation and in testbeds constructed of real devices. Shu et al. in [12] discuss the research directions for existing and future gas leakage source detection and boundary estimation schemes with wireless sensor networks. The authors of [13] present a survey of selected approaches for monitoring phenomena clouds. They can be classified concerning various criteria, such as a task to be performed, equipment used and expected network configuration, sensing and control strategies, communication and computing algorithms, and the accuracy of utilized gas dispersion models. A significant problem addressed in the literature is the resilience of the monitoring network to failures and insufficient energy resources. Imran and Ko in [14] present an energy-efficient method for detecting and estimating continuous objects spread over a large area by a failure-prone network. Improving the accuracy of boundary estimation while reducing the energy consumption is considered in [15].

This paper focuses on mobile sensing networks for phenomena-cloud boundary estimation and tracking. In general, we can distinguish simple systems built of a single mobile sensor or more complex systems comprised of a team of sensing devices. The simple systems for gas cloud boundary tracking are described in [16,17]. Wang et al. in [16] propose the algorithm for boundary estimation and tracking using a gas concentration gradient. A single moving platform equipped with the punctual sensor explores the sensing scene and constantly measures the gas concentration. The concentration gradient and consequently a new position of the platform are calculated based on these measurements. The algorithm for control the motion of a single unmanned underwater vehicle (UUV) equipped with sensors is presented and examined in [17]. Similarly to [16], both the initial detection of the cloud boundary and the tracking procedures are based on calculating the toxic substance concentration gradient. Moreover, the current speed measured by a dedicated sensor is taken into account in motion trajectory calculation. The proposed control algorithm was validated in the natural water reservoir with a dyeing substance imitating the toxic one. The paper [18] addresses the problem of boundary tracking for the mobile robot with uncertain dynamics and external disturbances. Sun et al. present and evaluate an adaptive control system using a radial basis function neural network to approximate a nonlinear function containing the uncertain model terms and toxic substance concentration gradient. Simulation results illustrate the stability of the system.

Another group of systems is networks built from cooperating or non-cooperating devices. The scheme for data gathering by multiple autonomous and non-cooperating underwater vehicles is described and discussed in [19]. In this approach, each device’s target position is determined autonomously. The current positions of other team members do not influence this device’s position calculation. A similar approach with multiple autonomous vehicles used to track boundaries is described in [20].

Many researchers address the problem of boundary tracking by a team of cooperating vehicles. Singh et al. in [21] describe a simple mechanism for unmanned vehicle cooperation. It is composed of two phases: first, mobile devices that form the network explore roughly the whole area of interest, then the initial shape of a boundary is discovered. In the second phase, each node follows the boundary to increase the accuracy of the initial estimation. Singh et al. demonstrate that their strategy can be easily adapted to a network consisting of multiple devices. In the case of a wide sensing area, it can be divided into subregions that are equally assigned to all nodes. Data collected by all nodes are gathered by a selected node that estimates the boundary’s shape. Next, the estimated boundary is divided into parts that are assigned to nodes, respectively, for further investigation. This approaches’ main drawback is that each device has to cover a long distance in the case of a vast cloud. Triandaf and Schwartz in [22] present a method for calculating the motion path for the formation of communicating sensors. The aim of all sensors is to follow a time-dependent concentration gradient. The algorithm allows the sensors to move in space in a non-stationary environment. It can be used for finding and tracking the boundary of any stable surface. The common feature of the solutions proposed in [23,24] is the concept of nominating a leader of the whole formation. This leader is selected from the group of devices located close to the cloud boundary. The objective of the sensing system described in [23] is to detect and track a wildfire border. It is assumed that network nodes measure the temperature of the air. The Rothermel model describing a spreading fire and the Kalman filter to detect a boundary in a sensing space are used to develop an algorithm for the motion control of all sensing devices. Unfortunately, because the Rothermel model is directly used to calculate all sensing devices’ target positions, this computing scheme can not be quickly adapted to other use cases. In [24], a team of four mobile robots is used to track the boundary of a phenomena cloud. A concentration gradient is used to calculate the target positions of all robots. At every time step, all devices equipped with punctual sensors measure the concentration of substances, and the concentration gradient is calculated. All devices’ target positions are calculated, solving the optimization problem with the performance function dependent on the concentration gradient. The application of algorithms for motion planning based on the concentration gradient and the concept of a virtual potential field [7], which are widely used in mobile robotics, is presented in [25]. Both techniques were verified and validated through simulation of the underwater oil spill tracking by unmanned underwater vehicles.

An alternative approach to tracking heavy gas clouds is to apply accurate models of the phenomena and use computer simulation to predict cloud dynamics and environmental risk. The heavy gas dispersion simulators SLAB [26] and Fluidyn-PANACHE [27] are commonly used. In [1], rapid assessment of exposure to chlorine released from a train derailment is described. However, to estimate the area covered by the heavy gas cloud, additional data and measurements have to be provided, i.e., landform, wind direction and strength, and obstacles presence. The quality of the cloud’s boundary estimation is usually sensitive to the accuracy of these data.

Reviewing the literature shows that most of the proposed gas cloud tracking systems built from mobile sensing devices use accurate gas concentrations in a sensing space and centralized schemes for node position calculation. The disadvantages of such an approach are discussed in Section 1. Therefore, our research focused on developing a solution that does not utilize the concentration gradient and implements the distributed mobility model.

## 3. Problem Formulation and Model of a Mobile Wireless Sensing Network

The aim is to design a sensing network composed of intelligent mobile wireless devices to explore an unknown environment to detect the toxic gas cloud, detect its boundaries, and finally, monitor and track the boundary of the evolving and moving cloud. Consider a network comprised by a set V of mobile sensing devices (network nodes) $D_{i}$, i=1,…,N

(1) G=(V,E),V≠∅,E≠∅,

where

(2) $$V=\{D_{i},i=1,\ldots ,N\},$$

(3) $$E=\{\left(D_{i},D_{j}\right):D_{i}\in V,D_{j}\in V,\;\;d_{j}^{i}\leq r_{t},\;\;i,j=1,\ldots ,N,\;\;i\neq j\}.$$

In the above formulas, E denotes the set of active direct connections between each pair of devices $D_{i}$, and $D_{j}$, i≠j, $\left(D_{i},D_{j}\right)$ is a bidirectional link. Each $D_{i}$ is equipped with the radio transceiver with radio range $r_{t}$ and a positioning system (GPS). Its position is described by a reference point $x_{i}=\left[x_{i_{1}},x_{i_{2}},x_{i_{3}}\right]$, which is the location of its antenna. $d_{j}^{i}=\left|\right|x_{i}-x_{j}\left|\right|$ is the Euclidean distance between $D_{i}$ and $D_{j}$. Assume that each node $D_{i}$ can freely change both its position and role in a network according to its knowledge about the environment and the network topology.

Each $D_{i}$ uses a punctual sensor to measure the gas concentration *g* at a given point in a workspace *W* every regular time interval Δt. For the sake of simplicity, it is assumed that each sensor at time *t* returns one of two values

- g(x,t)=1: the gas concentration exceeds the threshold value $\bar{g}$,
- g(x,t)=0: otherwise.

This approach does not use a concentration gradient. Therefore, local disturbances in gas concentration can be ignored.

Each $D_{i}$ can move with the speed $v_{i}\in \left[v_{i_{min}},v_{i_{max}}\right]$ in a desirable direction. The common direction in motion planning is to apply an artificial potential function *V* that can be viewed as a landscape where the device moves from a high-value state to a low-value state. A value of this function can be viewed as energy and its gradient as a force. *V* can be constructed as a sum of repulsive and attractive potentials, i.e., $V\left(d\right)=V_{-}\left(d\right)+V_{+}$, where *d* denotes a distance between a given device and other devices in a network or obstacles in the working space. The meaning of $V_{-}\left(d\right)$ and $V_{+}$ is straightforward; the obstacle repels the device, the target point attracts it. Hence, the sum of both these influences draws the device to the target position while deflecting it from obstacles.

In our research, we have adopted a network connectivity-maintaining, virtual force mobility model described in [28] to calculate motion trajectories of platforms carrying sensors. In this model, a simple artificial potential function drawing on the Lennard–Jones potential used in liquid crystals is applied to model the interactions between moving devices and their displacement calculation. The function $V_{j}^{i}$ that models the interactions between two devices $D_{i}$ and $D_{j}$ is as follows

(4) $$V_{j}^{i}\left(d_{j}^{i}\right)={\left(\frac{{\bar{d}}_{j}^{i}}{d_{j}^{i}}-1\right)}^{2},\;d_{j}^{i}=\left|\right|x_{i}-x_{j}\left|\right|,$$

where ${\bar{d}}_{j}^{i}$ denotes the reference distance between $D_{i}$ and $D_{j}$. The total potential between $D_{i}$ and all other *m* objects in the working space, i.e., other sensing devices and obstacles in the working space and target position, can be expressed as follows

(5) $$V_{j}^{i}\left(d_{1}^{i},d_{2}^{i},\ldots ,d_{m}^{i}\right)=\sum_{j=1}^{m}\epsilon_{j}^{i}V_{j}^{i}\left(d_{j}^{i}\right).$$

where $\epsilon_{j}^{i}>0$ denotes the weighting factor determining the importance of the impact of the object *j* on $D_{i}$. Moreover, since the external communication system can be broken or suffer from congestion in the disaster area, it is assumed that the distance between the selected, critical pairs of nodes should not be greater than the specified safe distance that guarantees permanent connectivity in a network.

The task is to calculate the optimal positions of all $D_{i}$, i=1,…,N at given time steps for a given application scenario. From Equation (4), it is obvious that in the reference positions of all $D_{i}$, namely such that ${\bar{d}}_{j}^{i}=d_{j}^{i}$, an optimal network topology is obtained.

## 4. An Overview of a Method for Heavy Gas Cloud Monitoring and Tracking

**Figure 1:**
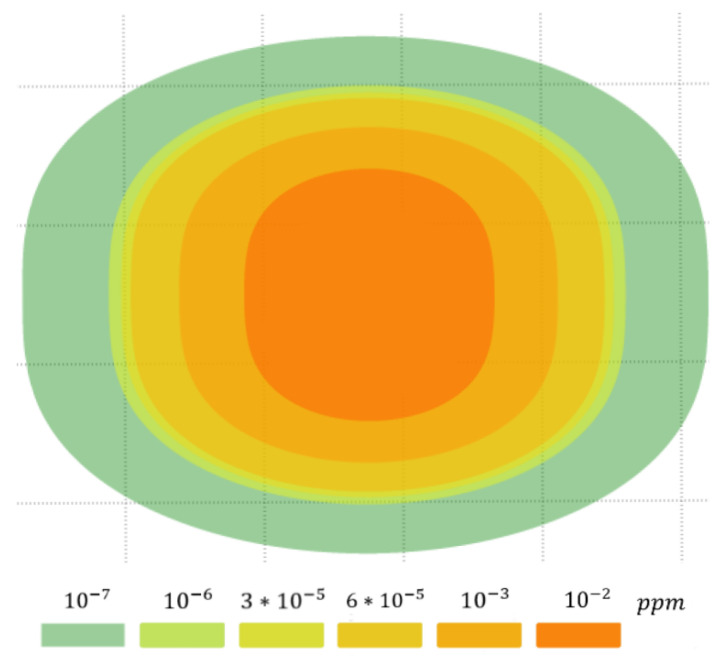
Typical characteristics of a heavy gas cloud.

The application scenario considered in this paper is heavy gas cloud detecting and monitoring. Figure 1 shows a typical heavy gas cloud. Due to its dispersion characteristics, heavy gas forms a cloud with the largest cross-section at its base.

In situational awareness tasks, determining the extent of contamination and delineating the area at risk is critical. Moreover, in most heavy gas propagation simulators, the cloud moves over a flat area and ignores local terrain and obstacles, which also cause changes in gas density. Therefore, although our virtual force motion model allows motion trajectories to be determined in three-dimensional space, the research presented in this paper focuses on the two-dimensional and obstacle-free workspace.

Consider the self-organizing network defined in Equations (1)–(5). Assume the sensing devices (network nodes) $D_{i}$ (i=1,…,N) do not have any information about the environment, particularly the cloud location. However, they can exchange data with each other about their positions and the measured gas concentrations. Based on this information, they autonomously determine the direction and speed of movement in a workspace. The limitation of the number of sensor readings g(x,t) to two values (0 and 1) allows fast detection of the cloud boundary. Changing the reading value indicates crossing the boundary.

Our heavy gas cloud monitoring method is composed of three main steps executed sequentially.

- Stage 1: workspace exploration to search for a gas cloud.
- Stage 2: cloud exploration to detect its boundary.
- Stage 3: permanent exploring and tracking of a cloud boundary.

All network nodes perform at regular intervals Δt of gas concentration measurements and exchange information about their locations and current sensors readings. Then, based on the obtained data, an optimal new target position of each device is calculated.

### 4.1. Stage I: Gas Cloud Detection

A team of *N* sensing devices $D_{i}$, i=1,…,N is used to explore the sensing scene *W* to search for a gas cloud. The device $D_{1}$ is responsible for maintaining the connectivity with the base station (BS)—the system’s central station. Other devices enable constant communication with $D_{1}$ using multi-hop transmission. Hence, all $D_{i}$, i=1,…,N create the coherent searching network that maintains continuous connectivity with BS.

The first stage begins with setting the target point c in the workspace. It is randomly selected. The device closest to the point c is elected to be a temporary leader $D_{leader}$ of the team. $D_{leader}$ is forced to move in the direction of c, the other nodes are forced to follow $D_{leader}$. A new position of $D_{leader}$ is calculated every Δt solving the following optimization problem

(6) $$\min_{x_{i}}\left[V^{i}={\left(\frac{{\bar{d}}_{c}^{i}}{d_{c}^{i}}-1\right)}^{2}\right],\;d_{c}^{i}=||c-x_{i}\left|\right|,$$

with reference distance ${\bar{d}}_{c}^{i}>0$ closed to 0. Other devices follow the $D_{leader}$. They move in formation. The process is repeated until the cloud is detected by at least one sensing device $D_{k}\in V$. Then $D_{k}$ continues its movement, and the remaining devices are attracted to $D_{k}$. Their displacements are calculated as follows

(7) $$\min_{x_{i}}\left[V^{i}={\left(\frac{{\bar{d}}_{k}^{i}}{d_{k}^{i}}-1\right)}^{2}\right],\;i=1,\ldots ,N,\;i\neq k,$$

with positive reference distance ${\bar{d}}_{k}^{i}\approx 0$.

**Figure 2:**
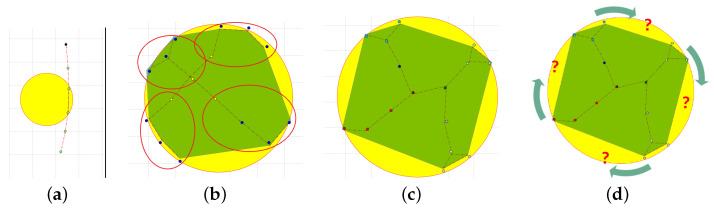
Gas cloud detection and monitoring: (**a**) Stage 1 completed (**b**) Stage 2: clusters (M=4) and temporal topology, (**c**) Stage 2 completed, (**d**) Stage 3: potential direction of boundary tracking.

The cloud can move and change its shape over time. All $D_{i}$ measure the gas concentration every Δt and in the case of $g(x_{i},t)=0$, they move towards the cloud. The first stage ends when sensors of at least two devices detect gas concentration higher than $\bar{g}$ (Figure 2a).

(8) $$\exists_{\left(i,j\right),i\in \left\{1,\cdots ,N\right\},j\in \left\{1,\cdots ,N\right\},i\neq j}:g\left(x_{i},t\right)=1\wedge g\left(x_{j},t\right)=1.$$

Then, the location Ψ of the gas cloud center is estimated based on data received from devices located inside the cloud.

(9) $$\Psi =\frac{\sum_{D_{i}\in V^{+}}x_{i}}{|V^{+}|},$$

where $V^{+}$ denotes a set of devices located inside the cloud. The number of devices with positive sensor readings increases in time. Ψ is updated based on new data received from all these devices.

### 4.2. Stage 2: Gas Cloud Boundary Detection

The second stage aims to extract the cloud from the environment, namely, detect its boundary, not explore its inside. The boundary is defined as a curve dividing the workspace *W* into two regions, respectively, with a gas concentration greater and lower than a given concentration threshold $\hat{g}$. The optimal solution of Stage 2 is an even coverage of the cloud boundary with sensors. Hence, the target positions of all devices should be on the cloud boundary. To solve this task, we have to elbow sensing nodes, repulse them from the center of the cloud Ψ, and force them to move and take the target positions. The complexity of the problem grows in the case of vast clouds and an insufficient number of sensing devices. We developed and validated through simulations two computing schemes, centralized and distributed ones. In the centralized method, one node in a network is nominated for a network head $D_{H}\in V$. Each device $D_{i}$, i=1,…,N repetitively calculates its target position in *W*, solving the optimization problem

(10) $$\min_{x_{i}}\left[V^{i}=\epsilon_{\Psi }^{i}V_{\Psi }^{i}+\sum_{D_{j}\in N_{i},j\neq i}\epsilon_{j}^{i}V_{j}^{i}=\epsilon_{\Psi }^{i}{\left(\frac{{\bar{d}}_{\Psi }^{i}}{d_{\Psi }^{i}}-1\right)}^{2}+\sum_{D_{j}\in N_{i},j\neq i}\epsilon_{j}^{i}{\left(\frac{{\bar{d}}_{j}^{i}}{d_{j}^{i}}-1\right)}^{2}\right],$$

under the constraint

(11) $${\bar{d}}_{j}^{i}<r_{t}.$$

In the above formulas, $\epsilon_{\Psi }^{i}\geq 0$ and $\epsilon_{j}^{i}\geq 0$ denote weighting factors determining the importance of the impact of, respectively, the center of the cloud Ψ and the neighboring node $D_{j}$ on the new position of $D_{i}$. $d_{\Psi }^{i}$ and $d_{j}^{i}$ are real Euclidean distances between $x_{i}$ and, respectively, Ψ and $x_{j}$ after a network transformation. ${\bar{d}}_{\Psi }^{i}$ and ${\bar{d}}_{j}^{i}$ are the reference distances between $x_{i}$ and, respectively, Ψ and $x_{j}$. These distances are repetitively calculated by the leader of a network $D_{H}$. $N_{i}=\{\left(D_{i},D_{n}\right):||x_{i}-x_{n}\left|\right|\leq r_{t}\},n=1,\ldots ,N$ is a set of neighboring nodes of the node $D_{i}$ and $r_{t}$ is the radio range.

In contrast to the centralized method, a clustering-based technique is proposed. The sensing network G (1) is divided into *M* separated subnetworks (clusters of devices) $V_{m}$, m=1,…,M, $V_{1}\cup V_{2}\cup \ldots \cup V_{M}=V$, $V_{1}\cap V_{2}\cap \ldots \cap V_{M}=\emptyset$. In each cluster $V_{m}$, m=1,…,M one node is nominated for a cluster head $D_{H_{m}}\in V_{m}$. Moreover, we select one cluster head to be the head of the whole network, $D_{H}\in \left\{D_{H_{1}},\ldots ,D_{H_{M}}\right\}$. All cluster heads are responsible for maintaining permanent connectivity with $D_{H}$. All members of a given cluster have to maintain permanent connectivity with its cluster head. Hence, each network node repetitively calculates its target position in *W*, solving the optimization problem

(12) $$\begin{array}{c} \min_{x_{i}}\left[V^{i}=\epsilon_{\Psi }^{i}V_{\Psi }^{i}+\sum_{D_{j}\in N_{i},D_{j}\in V_{m}}\epsilon_{j}^{i}V_{j}^{i}+\sum_{k\in IC_{m}}\epsilon_{k}^{i}V_{k}^{i}\right.\\ =\left.\epsilon_{\Psi }^{i}{\left(\frac{{\bar{d}}_{\Psi }^{i}}{d_{\Psi }^{i}}-1\right)}^{2}+\sum_{D_{j}\in N_{i},D_{j}\in V_{m}}\epsilon_{j}^{i}{\left(\frac{{\bar{d}}_{j}^{i}}{d_{j}^{i}}-1\right)}^{2}+\sum_{k\in IC_{m}}\epsilon_{k}^{i}{\left(\frac{{\bar{d}}_{k}^{i}}{d_{k}^{i}}-1\right)}^{2}\right], \end{array}$$

where

(13) $${\bar{d}}_{k}^{i}=\frac{\sum_{k\in IC_{m}}d_{k}^{i}}{2}+w_{2},\;w_{2}>0.$$

In the above formulas, $\epsilon_{k}^{i}$ denotes a weighting factor determining the importance of the impact of the centroid of a cluster $V_{k}$, $d_{k}^{i}$ an actual Euclidean distance between $x_{i}$ and a centroid of *k*-th cluster ($c_{k}$). $IC_{m}$ denotes a set of indexes of two clusters closest to the *m*-th cluster that contains $D_{i}$. ${\bar{d}}_{k}^{i}$ is an average distance between centroids of two clusters with indexes from $IC_{m}$ increased by a slight distance margin $w_{2}$.

The calculation scheme and algorithm for selecting clusters for the set $IC_{m}$ are described in detail in [11]. Moreover, the authors of [11] present the results of the comparative study of two computing schemes: centralized and distributed. After numerous simulations, some improvements to the cluster-based algorithm that speed up the target positions calculations and do not affect the final result were introduced. Since the task of cluster leaders $D_{H_{m}}$, m=1,…,M is limited to maintaining communication, the optimization problem (12) that has to be solved by each $D_{H_{m}}$ can be simplified to the following one

(14) $$\begin{array}{c} \min_{x_{m}}\left[V^{m}=\sum_{D_{j}\in N_{m},D_{j}\in V_{m}}\epsilon_{j}^{m}V_{j}^{m}+\sum_{k\in IC_{m}}\epsilon_{k}^{m}V_{k}^{m}\right.\\ =\left.\sum_{D_{j}\in N_{m},D_{j}\in V_{m}}\epsilon_{j}^{m}{\left(\frac{{\bar{d}}_{j}^{m}}{d_{j}^{m}}-1\right)}^{2}+\sum_{k\in IC_{m}}\epsilon_{k}^{m}{\left(\frac{{\bar{d}}_{k}^{m}}{d_{k}^{m}}-1\right)}^{2}\right]. \end{array}$$

A sample result of the clustering-based algorithm is depicted in Figure 2b. The paper [29] defines criteria for sensing network topology assessment and describes the algorithm that can be used to temporarily detect optimal topology and complete the second stage of the computing scheme. In general, *stage 2* is completed when devices are almost evenly distributed on the boundary of the cloud, Figure 2c. The set of these devices’ locations can be used to discover a given gas cloud boundary’s shape.

### 4.3. Stage 3: Gas Cloud Boundary Tracking

The final aim is to explore and track the gas cloud and report changes to its boundary over time. Let us assume that both nodes and clusters are almost evenly dispersed on the area covered by the phenomena; Figure 2c and Stage 3 starts. Each cluster head $D_{H_{m}}$, m=1,…,M selects one node from *m*-th cluster members. Let us denote it $D_{P_{m}}$, where $D_{P_{m}}\in V_{m}$, and *m* is a cluster number. This node aims to move along the boundary over time to discover its current shape, Figure 2d. Other members of cluster *m* follow $D_{P_{m}}$, m=1,…,M. They serve as a communication link to the network head $D_{H}$, which collects data from all network nodes.

#### 4.3.1. Node $D_{P_{m}}$ Selection

**Figure 3:**
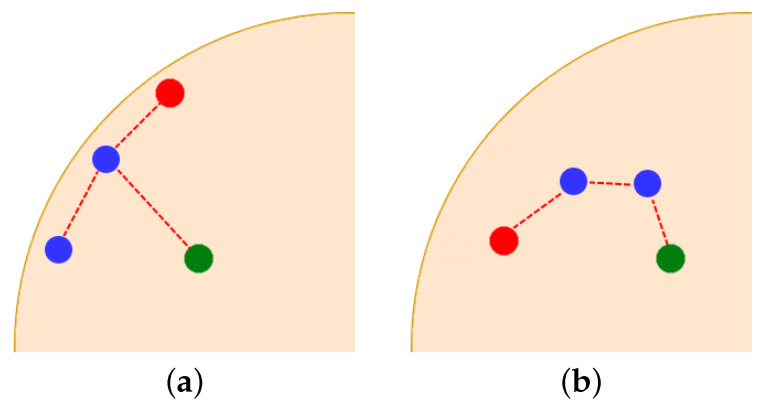
$D_{P_{m}}$ selection procedure (green node—$D_{H_{m}}$, red node—$D_{P_{m}}$): (**a**) $D_{P_{m}}$ selected among devices located on the boundary, (**b**) $D_{P_{m}}$ selected among devices located inside the cloud.

Let us consider the cluster $V_{m}$. One can distinguish two situations (Figure 3). Some members of the *m*-th cluster are located on a boundary (Figure 3a). The set of nodes located on a boundary at time *t* is defined as follows

(15) $$\{D_{i}\in V_{m}:\exists_{t^{*}\in \left[t-\Delta t_{out},t\right]}g\left(x_{i},t^{*}\right)=0\},$$

where $\Delta t_{out}$ denotes a fixed time period. The node most advanced in a clockwise direction is selected to play the role of $D_{P_{m}}$.

Otherwise, when all cluster *m*-th members are located inside the cloud, the node furthest from the cluster head $D_{H_{m}}$ is nominated for $D_{P_{m}}$ (Figure 3b). The distance between a given node $D_{i}\in V_{m}$ and $D_{H_{m}}$ is calculated in hops, i.e., number of relay nodes.

#### 4.3.2. Motion Trajectory Calculation

Both $D_{P_{m}}$, m=1,…,M and other members of clusters repetitively calculate their target positions in *W*, solving the optimization problems with performance measures defined respectively to their roles in the sensing network.

**Figure 4:**
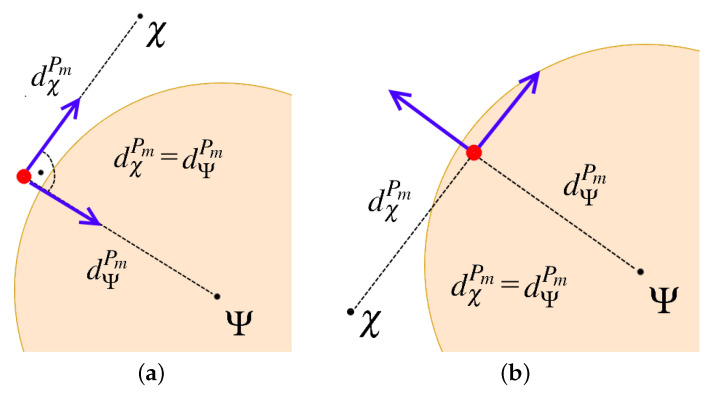
The location of the point χ: (**a**) $D_{P_{m}}$ (red dot) outside the gas cloud, (**b**) $D_{P_{m}}$ inside the gas cloud. The artificial potential forces $V_{\Psi }^{P_{m}}$ and $V_{\chi }^{P_{m}}$—blue arrows.

Let us start from the leaders of tracking teams $D_{P_{m}}$, m=1,…,M displacements calculation. Two points that influence these nodes’ motion trajectories can be defined, i.e., centroid of the cloud Ψ (9) and the point χ⊂W that forces the node to follow the cloud boundary. Hence, we obtain two functions $V_{\Psi }^{P_{m}}$ and $V_{\chi }^{P_{m}}$ that model the interactions between each $D_{P_{m}}$ and, respectively, Ψ and χ. The method for determining point χ is shown in Figure 4. Two cases are considered, the first one with the current position of $D_{P_{m}}$ outside the cloud (Figure 4a) and the second one with $D_{P_{m}}$ inside the cloud (Figure 4b).

Finally, a new position of $D_{P_{m}}$ is calculated every Δt, solving the following optimization problem.

(16) $$\begin{array}{c} \min_{x_{P_{m}}}\left[V^{P_{m}}=\epsilon_{\Psi }V_{\Psi }^{P_{m}}+\epsilon_{\chi }V_{\chi }^{P_{m}}\right.\\ =\left.\epsilon_{\Psi }{\left(\frac{{\bar{d}}_{\Psi }^{P_{m}}}{d_{\Psi }^{P_{m}}}-1\right)}^{2}+\epsilon_{\chi }{\left(\frac{{\bar{d}}_{\chi }^{P_{m}}}{d_{\chi }^{P_{m}}}-1\right)}^{2}\right], \end{array}$$

where $d_{\Psi }^{P_{m}}$ and $d_{\chi }^{P_{m}}$ denote an actual Euclidean distances between $D_{P_{m}}$ and, respectively, the estimated centroid of the cloud Ψ and point χ. ${\bar{d}}_{\Psi }^{P_{m}}$ and ${\bar{d}}_{\chi }^{P_{m}}$ are reference distances between $D_{P_{m}}$ and points Ψ and χ. Note that the cloud is in motion. Each node $D_{P_{m}}$, m=1,…,M is forced to move inside or outside the cloud depending on the sensor reading. Hence, the motion trajectories oscillate around the cloud boundary. To force oscillations, the reference distances ${\bar{d}}_{\Psi }^{P_{m}}$ in Equation (16) are defined as follows

(17) $${\bar{d}}_{\Psi }^{P_{m}}=\left\{\begin{array}{cc} \max_{D_{i}\in V^{+}}d_{\Psi }^{i}+w_{1} & \mathrm{if}\;g(x_{P_{m}},t)=1,\\ \min_{D_{i}\in V^{+}}d_{\Psi }^{i} & \mathrm{if}\;g(x_{P_{m}},t)=0, \end{array}\right.$$

where $V^{+}$ denotes the set of devices located inside the cloud. Other members of the clusters $D_{i}\in V_{m}$, m=1,…,M (including $D_{H_{m}}$) aim to maintain connectivity between nodes $D_{P_{m}}$ and $D_{H}$. They are relay nodes in communication between $D_{P_{m}}$ and $D_{H}$. It is evident that each node $D_{i}\in V_{m}$, $D_{i}\neq D_{P_{m}}$ is forced to follow $D_{P_{m}}$. Hence, its new position is calculated every Δt, solving the optimization problem.

(18) $$\min_{x_{i}}\left[V^{i}=V_{P_{m}}^{i}={\left(\frac{{\bar{d}}_{P_{m}}^{i}}{d_{P_{m}}^{i}}-1\right)}^{2}\right],\;{\bar{d}}_{P_{m}}^{i}\leq r_{t},$$

where $d_{P_{m}}^{i}$ denotes an Euclidean distance between $D_{i}\in V_{m}$ and $D_{P_{m}}$, ${\bar{d}}_{P_{m}}^{i}$ is a reference distance between $D_{i}$ and $D_{P_{m}}$. $r_{t}$ is the radio transmission range.

#### 4.3.3. Weighting Factor Calculation

**Figure 5:**
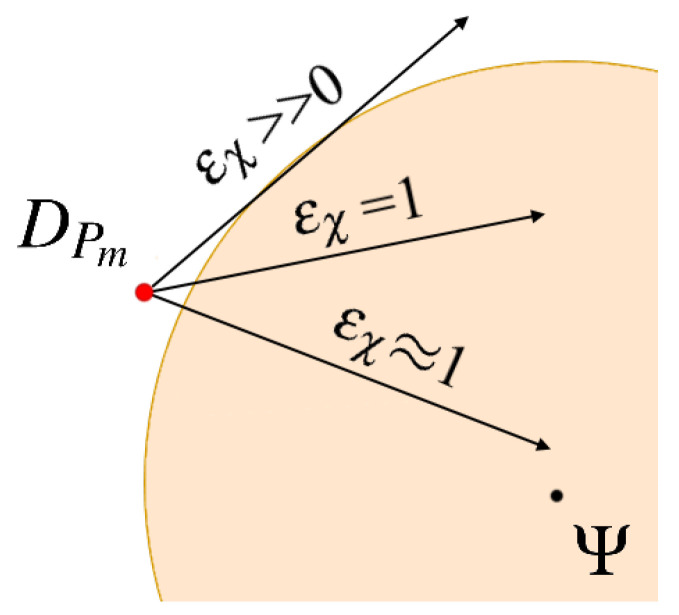
The impact of the coefficient $\epsilon_{\chi }$ on the motion direction of the node $D_{P_{m}}$.

Several parameters have to be identified in the optimization task (16). To simplify the model, we assumed without loss of generality ${\bar{d}}^{P_{m}}={\bar{d}}_{\chi }^{P_{m}}={\bar{d}}_{\Psi }^{P_{m}}$ and $\epsilon_{\Psi }=1$. Hence, the optimization problem (16) is reduced to the following one

(19) $$\min_{x_{P_{m}}}\left[V^{P_{m}}=V_{\Psi }^{P_{m}}+\epsilon_{\chi }V_{\chi }^{P_{m}}\right.=\left.{\left(\frac{{\bar{d}}^{P_{m}}}{d_{\Psi }^{P_{m}}}-1\right)}^{2}+\epsilon_{\chi }{\left(\frac{{\bar{d}}^{P_{m}}}{d_{\chi }^{P_{m}}}-1\right)}^{2}\right],$$

and only coefficient $\epsilon_{\chi }$ must be tuned. The impact of $\epsilon_{\chi }$ on the recommended motion direction of the node $D_{P_{m}}$ is depicted in Figure 5.

**Figure 6:**
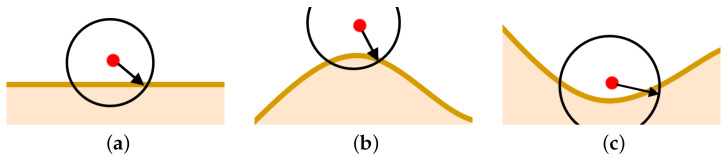
Recommended directions of $D_{P_{m}}$ displacements; various shapes of boundaries (**a**) flat; (**b**) convex; (**c**) concave.

The recommended motion direction should be fit to the shape of a given boundary. Figure 6 presents the examples of various shapes of the cloud boundary and adequate directions of $D_{P_{m}}$ displacements.

Assume $\tau_{z}$, z=1,2,…, denote time stamps of $D_{Pm}$ detector state changes ($g(x_{P_{m}},\tau_{z})$$\neq g(x_{P_{m}},\tau_{z}-\Delta t)$) and $\tau_{z+1}-\tau_{z}=k_{z}\cdot \Delta t$, where $k_{z}$ is a number of sensor readings taken since the last change of the detector state. Five methods for the weighting parameter $\epsilon_{\chi }$ in Equation (19) updating every Δt, i.e., at time steps $t=\tau_{z}+\Delta t,\tau_{z}+2\Delta t,\ldots ,\tau_{z}+\left(k_{z}-1\right)\Delta t$, z=1,2,…, were proposed and tested. The input data contains a list of historical values of $\epsilon_{\chi }$ and a list of historical bivalent sensor readings. The following items present the algorithms for adjusting the factor in the periods between subsequent sensor readings changes, i.e., $\tau_{z+1}-\tau_{z}$, z=1,2,…,.

***Variant*** ***I*** A constant value of the weighting factor in the whole tracking horizon. $\epsilon_{\chi }^{\left(k\right)}=\bar{\epsilon_{\chi }}$, $k=0,1,\ldots ,k_{z}$ where $\bar{\epsilon_{\chi }}$ is the predefined value, the same one regardless of the sensor state.
***Variant*** ***II*** Decreasing the value of the weighting factor over time (the closer the device to the cloud boundary, the slower it moves). The new value of $\epsilon_{\chi }$ is calculated based on the previous one, starting from the predefined value)
  (20) $$\epsilon_{\chi }^{\left(0\right)}={\bar{\epsilon }}_{\chi },\;\epsilon_{\chi }^{\left(k\right)}=l\cdot \epsilon_{\chi }^{\left(k-1\right)},\;k=1,2,\ldots ,k_{z},$$
  where $l\in \left(0,1\right)$.
***Variant*** ***III*** The initial value of $\epsilon_{\chi }$ for time stamps $\tau_{z}$, z≥3 is calculated as a linear combination of a predefined constant value and value of $\epsilon_{\chi }$ at the time stamp of the same change of sensor state in the past.
  (21) $$\epsilon_{\chi }^{\left(0\right)}=\left\{\begin{array}{cc} \bar{\epsilon_{\chi }}, & z<3,\\ \alpha \cdot \epsilon_{\chi }^{z-2}+\left(1-\alpha \right)\cdot {\bar{\epsilon }}_{\chi }, & z\geq 3, \end{array}\right.$$
  where α∈(0,1) is an experimentally adjusted model parameter and ${\bar{\epsilon }}_{\chi }$ a predefined constant value. $\epsilon_{\chi }^{z-2}$ is a value of the factor calculated at time stamp $\tau_{z-1}-\Delta t$. The scaling factor is decreased according to the Equation (20).
***Variant*** ***IV*** $\epsilon_{\chi }^{\left(0\right)}=\bar{\epsilon_{\chi }}$ at time stamps $\tau_{z}$, z<3. At time stamps $\tau_{z}$, z≥3 the initial value of $\epsilon_{\chi }$ is calculated according to the following formula
  (22) $$\epsilon_{\chi }^{\left(0\right)}=\left\{\begin{array}{cc} \epsilon_{\chi }^{z-2}, & k_{z-2}>1,\\ \epsilon_{\chi }^{z-2}+\bar{b}, & k_{z-2}=1, \end{array}\right.$$
  where $\tau_{z-1}-\tau_{z-2}=k_{z-2}\cdot \Delta t$, $\bar{b}>0$ is a constant value used to increase the speed of node $D_{P_{m}}$. The scaling factor is decreased according to the Equation (20).
***Variant*** ***V*** Modification of variant IV by replacing the fixed $\bar{b}$ with the variable *b*. This variable is updated every $\tau_{z}$, z=1,2,…. It is calculated as follows
  (23) $$b^{z}=\left\{\begin{array}{cc} \bar{b}, & k_{z-2}>1,\\ b^{z-2}+\Delta b, & k_{z-2}=1, \end{array}\right.$$
  where Δb>0 is a fixed value and $b^{z-2}$ a value of the variable *b* calculated at $\tau_{z-1}-\Delta t$. Finally, the initial value of the factor $\epsilon_{\chi }$ at time stamps $\tau_{z}$, z≥3 is calculated according to the following formula
  (24) $$\epsilon_{\chi }^{\left(0\right)}=\left\{\begin{array}{cc} \epsilon_{\chi }^{z-2}, & k_{z-2}>1,\\ \epsilon_{\chi }^{z-2}+b^{z}, & k_{z-2}=1. \end{array}\right.$$
  Next, the scaling factor is decreased according to the Equation (20).

### 4.4. Nonconvex Boundary Tracking

**Figure 7:**
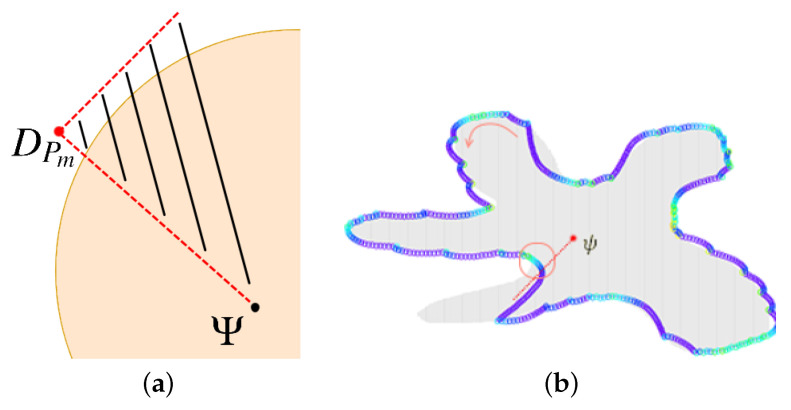
Tracking concave sections of the cloud boundary: (**a**) possible locations of target points, (**b**) sample motion trajectory of $D_{P_{m}}$ tracking a nonconvex cloud boundary.

The problem arises in the case of tracking the cloud boundary with concave sections. The possible target positions are limited to the quarter determined by the range of motion of $D_{P_{m}}$, Figure 7a. Therefore, it may take a long time to cover the border accurately, even if the value of factor $\epsilon_{\chi }$ is close to zero, (Figure 7b).

Hence, to improve the quality and speed up the concave sections tracking, the following correction procedure is proposed. In the case of $\epsilon_{\chi }<\epsilon_{min}$, where $\epsilon_{min}$ is a predefined threshold value, the point χ is relocated. Its position is replaced by a new one calculated by rotating the original one by the angle $r\frac{\pi }{2}$, $r\in \left\{-3,-2,-1,1,2,3\right\}$ in a clockwise direction relative to the position of $D_{P_{m}}$. Then, the optimization problem (19) is solved, and new positions of leaders of tracking teams $D_{P_{m}}$, m=1,…,M are calculated. The parameter *r* is calculated according to Algorithm 1.
**Algorithm 1** Detection of concave parts of boundary and *r* calculation1:r=02:**if**$\epsilon_{\chi }<\epsilon_{min}$ and $k_{z}>1$ **then**3: **if** $g(x_{P_{m}},t)=1$ **then**4:  r=r−15: **else**6:  r=r+17: **end if**8:**else if**$g\left(x_{P_{m}},t\right)\neq g\left(x_{P_{m}},t-\Delta t\right)$ and $k_{z-1}=1$ **then**9: **if** r<0 **then**10:  r=r+111: **end if**12: **if** r>0 **then**13:  r=r−114: **end if**15:**end if**

## 5. Experimental Study

The computing scheme for detecting and tracking heavy gas clouds was tested and evaluated through simulation. Many of the experiments were designed and conducted to tune the parameters of the developed algorithms for sensing devices’ motion planning and evaluate the efficiency of developed sensing networks. The sensing networks comprising intelligent wireless mobile devices were implemented in the MobASim simulation platform [30]. MobASim is a software environment for prototyping and simulation of wireless ad hoc networks in two-dimensional space. For the experiments, a simple box model of an instantaneous heavy gas cloud [31] that hazard analysts widely adopt was implemented. It provides a convenient and efficient method for atmospheric dispersion modeling.

### 5.1. Quality and Performance Metrics

Three metrics to measure the quality and efficiency of developed algorithms were defined, i.e., (i) the accuracy of the boundary estimation, (ii) the time used to discover the boundary, (iii) frequency of boundary-crossing.

Compact sensors’ coverage of the boundary of the cloud is the most crucial requirement. To evaluate this requirement, the widely used accuracy measure acc was proposed

(25) $$acc=\frac{TP+TN}{TP+TN+FN+FP}.$$

**Figure 8:**
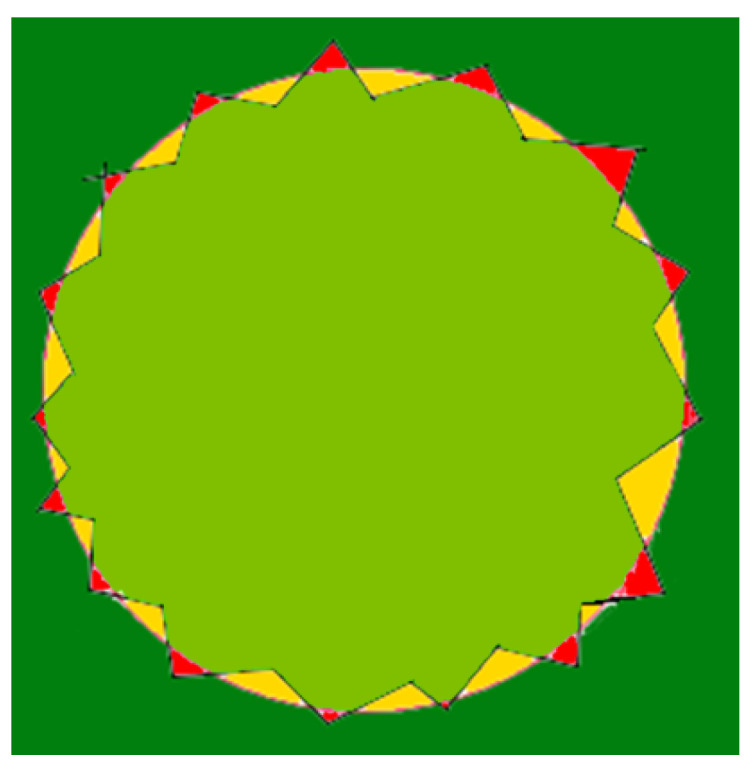
The cloud boundary approximation: TP (light green), TN (bottle green), FN (yellow) and FP (red).

Let us assume that $t_{circle}$ denotes a lap time of the gas cloud by the sensing device $D_{P_{m}}$. Subsequent target positions of $D_{P_{m}}$ are calculated every Δt. Hence $t_{circle}=L\cdot \Delta t$, where *L* is the number of calculated positions. Let us use linear interpolation. The input data set consists of *L* target positions of $D_{P_{m}}$. Finally, we can approximate the cloud boundary and use it to estimate the area covered by the cloud. Figure 8 shows the result for the circular gas cloud covering the part of the given working space. Then, we can calculate the values of TP (true positive), TN (true negative), FN (false negative) and FP (false positive) in Equation (25). TP and TN denote the correctly recognized area by our sensing devices, respectively, inside and outside the cloud, while FN and FP denote incorrectly recognized areas. It is evident that the greater the acc measure, the better the approximation of the boundary.

In general, it is desirable for the sensing device to cross the boundary as frequently as possible. It means that the devices are still within the narrow margin of the boundary. Hence, the corresponding measure $f_{crossing}$ can be defined

(26) $$f_{crossing}=\frac{\sum_{t=1}^{t_{circle}-1}h}{t_{circle}-1},\;h=\left\{\begin{array}{cc} 0 & g\left(x_{P_{m}},t\right)=g\left(x_{P_{m}},t+\Delta t\right)\\ 1 & g\left(x_{P_{m}},t\right)\neq g\left(x_{P_{m}},t+\Delta t\right) \end{array}\right.$$

The linear interpolation better approximates the boundary of the cloud when there is a higher $f_{crossing}$. Finally, a cloud boundary exploration time $t_{circle}$ should be as short as possible.

An indicator *q* that aggregates all described metrics has been introduced to simplify the sensing network performance evaluation

(27) $$q=\frac{{\left(f_{crossing}\right)}^{w_{f}}\cdot acc^{w_{acc}}}{{\left(t_{circle}\right)}^{w_{t}}},$$

where $w_{t}>0$, $w_{f}>0$ and $w_{acc}>0$ are parameters that indicate the importance of given measures.

### 5.2. Scenario Description

The testing scenario was a synthetic network comprised of 16 sensing devices designed to detect and track the chlorine cloud resulting from an immediate leak. All devices were equipped with radio transceivers with a maximum transmit power equal to −10 dBm (transmission range $r_{t}$ = 162.86 m), sensors measuring gas concentration every Δt = 1 s and moving platforms with a maximum speed 15 ms. In all experiments, the network was divided into four autonomous clusters. In general, the subnetworks trucked the cloud independently, and the only binding element was the center of cloud Ψ. The following values of parameters in indicator *q* (27) were fixed: $w_{t}=3$, $w_{f}=1$ and $w_{acc}=2$.

Five simulation scenarios were tested: *Cloud 1*, a gas cloud with a circle-shaped border, *Cloud 2*, a gas cloud with an ellipse-shaped border, *Cloud 3*, a gas cloud with a mixed border, partially circle-shaped and partially ellipse-shaped, and two gas clouds, i.e., *Cloud 4* and *Cloud 5*, with nonconvex borders. The average values of performance metrics acc, $f_{crossing}$, $t_{circle}$ and *q* calculated by leaders of all clusters are presented in the tables. All figures illustrate motion trajectories of device $D_{P_{m}}$ from the same selected cluster. Consecutive points show successive $D_{P_{m}}$ positions in the workspace (800 × 800 m). At this localization, $D_{P_{m}}$ measured the gas concentration. Colors indicate the values of $\epsilon_{\chi }$ used to calculate these points. Various shades of five colors: violet ($\epsilon_{\chi }\in$ [0.1, 2.5) ), blue ($\epsilon_{\chi }\in$ [2.5, 5.0)), green ($\epsilon_{\chi }\in$ [5.0, 7.5)), yellow ($\epsilon_{\chi }\in$ [7.5, 10)), red ($\epsilon_{\chi }\geq 10$) are used.

### 5.3. Model Parameters Tuning—Circle and Ellipse Shaped Clouds

**Figure 9:**
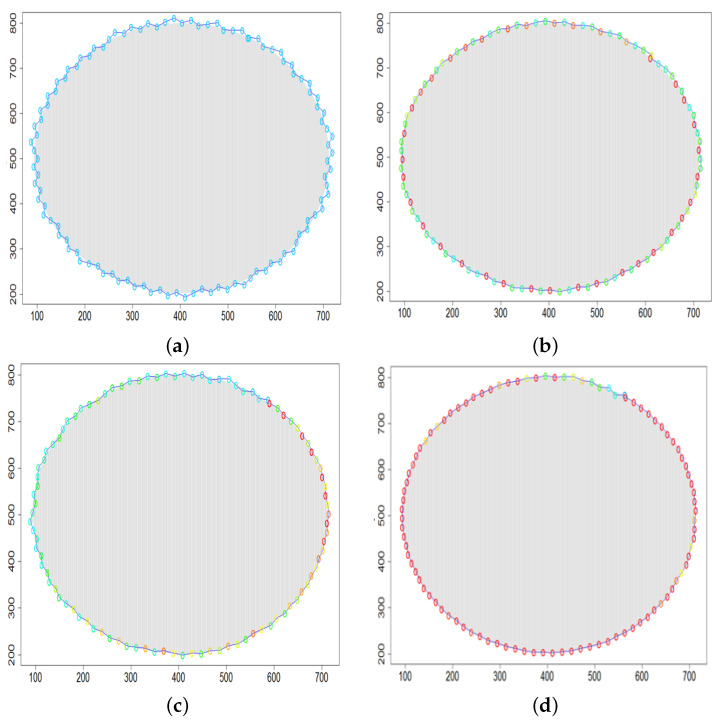
Successive $D_{P_{m}}$ positions during cloud boundary tracking calculated for four variants of $\epsilon_{\chi }$ updating procedures: (**a**) variant I, (**b**) variant III, (**c**) variant IV, (**d**) variant V; simulation scenario *Cloud 1*. The colors of the points correspond to different values of $\epsilon_{\chi }$.

**Figure 10:**
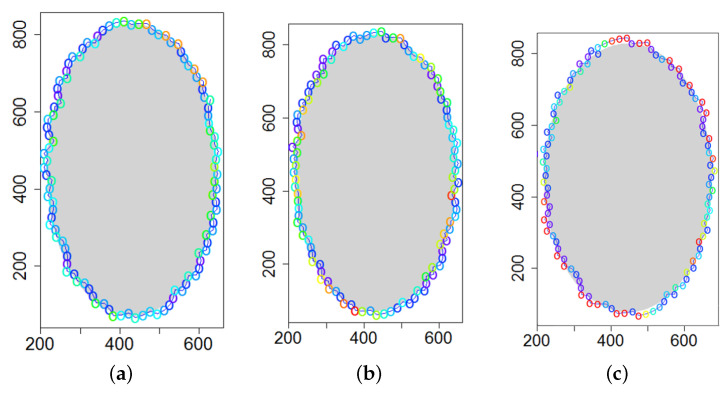
Successive $D_{P_{m}}$ positions during cloud boundary tracking calculated for three variants of $\epsilon_{\chi }$ updating procedures: (**a**) variant III, (**b**) variant IV, (**c**) variant V; simulation scenario *Cloud 2*. The colors of the points correspond to different values of $\epsilon_{\chi }$.

The first set of experiments were designed to examine and compare the performance and efficiency of the developed sensing network for various values of parameters ${\bar{\epsilon }}_{\chi }$, $\bar{b}$, Δb, *l* and α that have to be arbitrarily selected by the user and the weighting factor $\epsilon_{\chi }$ in Equation (19) calculated according to I-V variants described in Section 4.3.3. The aim was to test the sensitivity of the developed algorithm for boundary tracking of these parameters and to determine the values of all mentioned parameters and a variant of the $\epsilon_{\chi }$ updating procedure that guarantees high-quality boundary estimation. The results of simulations conducted for two different shapes of gas clouds, namely scenarios *Cloud 1* and *Cloud 2*, are presented in Table 1, Table 2, Table 3 and Table 4 and Figure 9 and Figure 10. Table 1 and Table 3 collect values of model parameters for which the highest value of the indicator *q* calculated for each variant of $\epsilon_{\chi }$ updating was obtained in both scenarios. Table 2 and Table 4 show the values of all metrics described in Section 5.1. The best ones are marked in green. Figure 9 and Figure 10 depict the motion trajectories (positions in time) of the selected device $D_{P_{m}}$ calculated for various variants of the $\epsilon_{\chi }$ updating procedure and circle-shaped and ellipse-shaped clouds.

It can be seen that, in general, the best values of all metrics were obtained for two variants of the weighting factor $\epsilon_{\chi }$ updating, namely IV and V for both simulation scenarios. However, variant IV was the best with respect to the indicator *q* aggregating all metrics, although the time used to circle the cloud was slightly longer than in the case of variant III. The optimal values of parameters ${\bar{\epsilon }}_{\chi }$ are lower for ellipse-shaped cloud boundary. A higher value of ${\bar{\epsilon }}_{\chi }$ increases the time for the device to return to the cloud region when the radius of the curvature of the cloud decreases rapidly. Moreover, it can be observed that each device needs more time to circle the ellipse-shaped cloud than the circle-shaped one. In general, tracking an ellipse-shaped cloud is a more difficult task.

### 5.4. Model Parameters Adjusting—Convex Clouds

The second set of experiments was designed to check how the model parameters tuned for networks tracking gas clouds with circle and ellipse-shaped boundaries can be adjusted to networks tracking gas clouds with a mixed border, partially circle shaped and partially ellipse-shaped (scenario *Cloud 3*). Two series of experiments were conducted. The aim was to compare the quality of boundary tracking in two cases (i) the weighting factor $\epsilon_{\chi }$ in Equation (19) calculated for values of parameters collected in Table 1, (ii) $\epsilon_{\chi }$ calculated for values of parameters collected in Table 3. The results are presented in Table 5 and Table 6.

As can be seen, all results of boundary tracking are, in general, acceptable. However, better accuracy of the boundary estimation was obtained using values of parameters tuned for the ellipse-shaped cloud. It is clearly illustrated in Table 6. Similar to previous experiments described in Section 5.3, the highest value of the indicator *q* was obtained for variant IV of the $\epsilon_{\chi }$ updating procedure. The time taken to circle the gas cloud was the shortest in the case of variant III. However, it should be noted that much worse results in comparison to those presented in Section 5.3 were achieved for variant V. It can be concluded that the presented approach is sensitive to model parameters. Hence, the number of parameters should not be too large as it is difficult to identify them for various shapes of clouds. Variant V can be successfully applied in tracking clouds with a big radius of curvature.

### 5.5. Model Parameters Adjusting—Nonconvex Clouds

**Figure 11:**
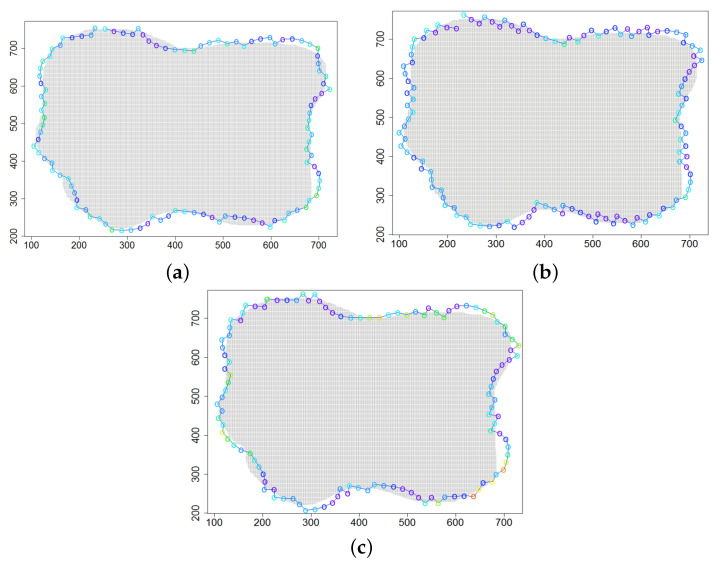
Successive $D_{P_{m}}$ positions during cloud boundary tracking calculated for three variants of $\epsilon_{\chi }$ updating procedures: (**a**) variant III, (**b**) variant IV, (**c**) variant V; simulation scenario *Cloud 4*. The colors of the points correspond to different values of $\epsilon_{\chi }$.

The experiments discussed in the two previous sections were conducted for convex clouds. Let us consider a nonconvex cloud with an irregular, nonconvex boundary, as presented in Figure 11, *Cloud 4*.

A series of simulations were conducted for three variants of the $\epsilon_{\chi }$ updating procedure and parameters from Table 1. Table 7 shows the values of metrics obtained for three variants of $\epsilon_{\chi }$ updating. Worse results were obtained than in scenarios with convex boundaries. It can be concluded that variant V of the $\epsilon_{\chi }$ updating procedure is suitable only when the boundary has a shape with a constant radius of curvature. In the case of two other variants, the results are very similar, and the decision to choose one of them requires prioritizing the criteria. When fast detection of the boundary is crucial, variant III is recommended. In addition, simulations were performed in which $\epsilon_{\chi }$ was calculated for parameters from Table 3. The results were a bit better, i.e., acc=0.962 and q=3.2.

**Figure 12:**
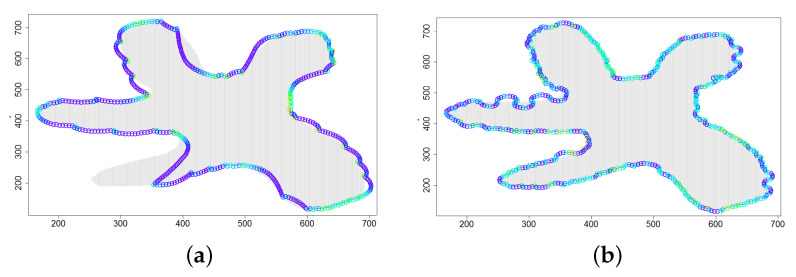
Successive $D_{P_{m}}$ positions during cloud boundary tracking calculated for two variants: (**a**) correction procedure disabled, (**b**) correction procedure enabled; simulation scenario *Cloud 5*. The colors of the points correspond to different values of $\epsilon_{\chi }$.

The last series of experiments aimed to test the efficiency of the correction procedure described in Section 4.4. The results of simulations for the application scenario *Cloud 5*, i.e., a cloud with multiple concave parts of its boundary, are presented in Table 8 and Figure 12. The weighting factor $\epsilon_{\chi }$ was calculated according to Equation (22) (variant IV) for values of parameters from Table 1. The threshold value $\epsilon_{min}$ was fixed to 0.3. The motion trajectory of the device $D_{P_{m}}$ presented in Figure 12b can be compared to the one calculated when the correction for concave parts of boundaries is disabled (Figure 12a). It can be seen that the correction procedure significantly improves the accuracy of cloud boundary estimation for very irregularly shaped clouds.

## 6. Conclusions

The paper summarizes the research results concerned with designing and developing distributed sensing systems for heavy gas cloud monitoring. This system comprises autonomous unmanned vehicles, equipped with punctual sensors, radio transceivers and GPS modules that spontaneously create a network of devices that adapt to achieve goals. The paper describes a three-stage strategy for boundary detection, estimation and tracking. In this approach, the optimal motion trajectories for all sensing devices’ discovering the current shape of the boundary are calculated based on the data collected from sensing vehicles and the models for target position calculation that incorporates artificial potential functions. The effectiveness was tested through extensive simulations. The presented results of experiments show that the design of an efficient sensing system for phenomena clouds should account for trade-offs between detection accuracy and computational complexity, measurement quality and equipment cost, communication reliability and network load. The challenge for the designer of this type of network is to develop efficient algorithms for online calculating of motion trajectories of sensing nodes and determine the optimal values of model parameters. This paper presents such an algorithm and examines the quality of various computing schemes for its parameter tuning. Simulation experiments showed the sensitivity of the proposed system to the model parameters and the need to select a procedure for tuning the coefficients of the optimization task to a particular scenario. Nevertheless, even for nonconvex clouds with irregular shapes, satisfactory results were obtained for calculated values of model parameters. Moreover, the simulation results demonstrated that using only a rough measurement to indicate that the threshold concentration value was exceeded can detect and track a gas cloud boundary. Calculating new sensor positions only based on the information about exceeding the safe gas concentration value makes our sensing system less sensitive to the quality of the gas concentration measurement. Because of the inaccuracy of actual sensors, such an approach may be better suited to real-world applications. Moreover, it can be easily adapted to detect other phenomena clouds, namely oil spill and wildfires, etc. To sum up, the simulation results corroborate our analysis and confirm that mobile ad hoc networks can be successfully used to monitor dynamic phenomena, create situation awareness and support rescue teams.

The research in that domain will be continued. The future work will focus on the experiments conducted on more complex and realistic scenarios with more detailed toxic gas dispersion models and workspace with obstacles. The goal is to test the sensitivity of our system on the speed of boundary evolvement and the impact of the obstacles on the quality of gas concentration measurement, radio communication and, consequently, the effectiveness of our monitoring system. Moreover, we plan to develop and investigate a version of the system to operate in a three-dimensional workspace. It will allow extending the possibilities of applications to monitor other phenomena such as oil spills, wildfire, moving groups of people.

## Figures and Tables

**Table 1:** Values of parameters used to calculate the optimal weighting factor $\epsilon_{\chi }$; indicator *q*, simulation scenario *Cloud 1*.

	Variant I	Variant II	Variant III	Variant IV	Variant V
${\bar{\epsilon }}_{\chi }$	2	5	10	2	2
*l*	-	0.8	0.8	0.8	0.8
α	-	-	0.9	-	-
$\bar{b}$	-	-	-	0.5	1
Δb	-	-	-	-	1.3

**Table 2:** Values of performance metrics obtained for five variants of $\epsilon_{\chi }$ updating; simulation scenario *Cloud 1*.

	Variant I	Variant II	Variant III	Variant IV	Variant V
$t_{circle}$	105.5	98.75	98.25	99.75	98.25
$f_{crossing}$	0.61	0.7	0.65	0.82	0.73
acc	0.977	0.988	0.988	0.99	0.991
*q*	4.9	7.0	6.7	8.1	7.6

**Table 3:** Values of parameters used to calculate the optimal weighting factor $\epsilon_{\chi }$; indicator *q*, simulation scenario *Cloud 2*.

	Variant I	Variant II	Variant III	Variant IV	Variant V
${\bar{\epsilon }}_{\chi }$	1	2	8	5	2
*l*	-	0.6	0.6	0.6	0.5
α	-	-	0.9	-	-
$\bar{b}$	-	-	-	0.9	1
Δb	-	-	-	-	1.3

**Table 4:** Values of performance metrics obtained for five variants of $\epsilon_{\chi }$ updating; simulation scenario *Cloud 2*.

	Variant I	Variant II	Variant III	Variant IV	Variant V
$t_{circle}$	107	111.5	106.5	108.75	108.5
$f_{crossing}$	0.45	0.56	0.57	0.65	0.64
acc	0.962	0.965	0.972	0.975	0.973
*q*	3.5	3.8	4.4	4.8	4.7

**Table 5:** Values of performance metrics obtained for five variants of $\epsilon_{\chi }$ updating and values of parameters from Table 1 and Table 3; simulation scenario *Cloud 3*.

	Variant I		Variant II		Variant III		Variant IV		Variant V	
	Table 1	Table 3	Table 1	Table 3	Table 1	Table 3	Table 1	Table 3	Table 1	Table 3
$t_{circle}$	116.5	149	112	125	112	116.5	116.5	117	113.75	122.5
$f_{crossing}$	0.52	0.81	0.37	0.73	0.54	0.69	0.68	0.72	0.52	0.69
acc	0.976	0.979	0.963	0.982	0.983	0.986	0.985	0.987	0.971	0.984
*q*	3.2	2.4	2.5	3.6	3.7	4.2	4.2	4.4	3.4	3.6

**Table 6:** Successive $D_{P_{m}}$ positions during cloud boundary tracking calculated for two variants of $\epsilon_{\chi }$ updating procedures and two sets of parameters from Table 1 and Table 3; simulation scenario *Cloud 3*.

Variant I		Variant II	
Table 1	Table 3	Table 1	Table 3
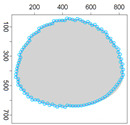	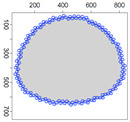	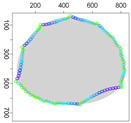	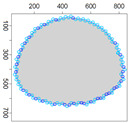
(**a**)	(**b**)	(**c**)	(**d**)
**Variant III**		**Variant IV**	
Table 1	Table 3	Table 1	Table 3
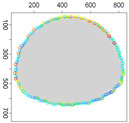	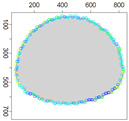	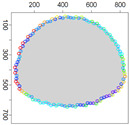	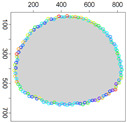
(**e**)	(**f**)	(**g**)	(**h**)
**Variant V**			
Table 1		Table 3	
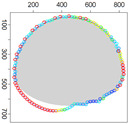		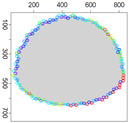	
(**i**)		(**j**)	

**Table 7:** Values of performance metrics obtained for three variants of $\epsilon_{\chi }$ updating; simulation scenario *Cloud 4*.

	Variant III	Variant IV	Variant V
$t_{circle}$	112	124	119.75
$f_{crossing}$	0.47	0.96	0.49
acc	0.956	0.991	0.949
*q*	3	2.9	2.6

**Table 8:** Values of performance metrics obtained for variant IV, parameters from Table 1, disabled and enabled correction procedure.

	Disabled	Enabled
$t_{circle}$	343.5	360.25
$f_{crossing}$	0.51	0.6
acc	0.987	0.99
*q*	$6.9\times 10^{-10}$	$8\times 10^{-10}$

## Data Availability

Not applicable.

## Appendix A. Summary of Standard Notation

G=(V,E)	a sensing network; V—a set of sensing devices, E a set of active direct connections between each pair of devices
$D_{i}$, $x_{i}$, $v_{i}$	*i*-th sensing device, its position in the workspace *W* and speed
$N_{i}$	a set of nodes in the radio range $r_{t}$ of the node $D_{i}$
$d_{a}^{i}$, ${\bar{d}}_{a}^{i}$	an actual Euclidean distance and a reference distance between $D_{i}$ and *a*
$\epsilon_{b}^{i}$	a weighting factor determining the importance of the impact of *b* on the new position of $D_{i}$
$V_{a}^{b}$	a virtual force that models the interactions between two points in the workspace *W*
g(x,t)	a gas concentration measured at point x and time *t*
$D_{leader}$	a formation leader in the gas cloud detection stage
$D_{H}$	a network head in the gas cloud boundary detection stage
$V_{m}$, $D_{H_{m}}$	*m*-th cluster and its cluster head
$D_{P_{m}}$	a leader of tracking team in *m*-th cluster (cloud boundary tracking stage)
Ψ, $\epsilon_{\Psi }$	an estimated gas cloud center and its weighting factor
χ, $\epsilon_{\chi }$	a point forcing a device to follow the cloud boundary and its weighting factor
$t_{circle}$, $f_{crossing}$, acc, *q*	quality metrics: a cloud boundary exploration time, a number of boundary crossing, an accuracy of boundary detection, an aggregated measure
